# Supplementary material for: Predictors of Urgent Cancer Care Clinic and Emergency Department Visits for Individuals Diagnosed with Cancer
Source: Curr Oncol. 2021 May 8;28(3):1773–89. doi: 10.3390/curroncol28030165 (PMC8161790; doi:10.3390/curroncol28030165)
Supplement: Supplementary file 1 [file curroncol-28-00165-s001.zip › curroncol-1179738-supplementary.pdf]

Supplemental Data

Table S1. List of ED visits excluded from the analyses

Type of visit	
Injury	Noxious inhalation
Trauma	Sting
Foreign body	Exposure to communicable disease
Laceration	Sexual assault
Exposure	Abuse
Abrasion	Chemical
Amputation	Postpartum bleeding
Bite	Community exposure
Booked elective	Ring removal
Burn	Cast check
Congenital problems in children	Contusion
Direct referral	Deformity
Neonatal jaundice	Inhalation
Newly born	Needle stick
Pregnancy	Possible SARS
Puncture wound	

Table S2. Hours between triage and discharge for individuals who visited the UCC or an ED by CTAS score, 2013-2016, Winnipeg, Manitoba (N=13,252 visits)

	UCC			ED		
CTAS score	1-2	3-5	Unknown	1-2	3-5	Unknown
Number of visits	683	2252	217	2611	7468	21
Mean (SD)	3 (1.5)	2 (1.5)	1 (1.3)	15 (14.4)	14 (14.9)	10 (18.0)
Median (IQR)	3 (2-4)	2 (1-3)	1 (0-1)	10 (6-21)	8 (5-19)	3 (1-11)

Abbreviations: UCC, urgent cancer care; ED, emergency department; CTAS, Canadian Triage Acuity Score; SD, standard deviation; IQR, interquartile range.

Table S3. Percent change in scaled Brier scores from full model for UCC and ED visits by variable and follow-up period

	Follow-up period, % change							
	1 to 6 months		7 to 12 months		13 to 18 months		19 to 24 months	
Variable	UCC	ED	UCC	ED	UCC	ED	UCC	ED
Chemotherapy	75.0	1.7	63.3	6.1	57.5	3.1	93.0	6.3
Radiation therapy	2.4	2.1	0.9	0.9	3.8	0.8	13.2	2.4
Hormone therapy	3.6	2.0	3.9	3.9	2.8	-1.0	2.3	0.8
Immunotherapy	0.9	0.7	-0.1	0.7	-4.3	0.2	13.3	0.1
Cancer site	11.0	4.8	15.4	0.9	0.5	6.2	5.5	11.5
Stage	0.6	13.4	2.9	9.5	5.4	9.6	3.7	1.3
Age	4.6	0.1	2.8	0.0	5.5	-4.2	6.9	-0.6
Sex	-0.1	-0.1	1.7	2.5	3.0	1.4	-0.6	0.1
Income quintile	1.9	-0.1	0.4	5.8	-0.7	0.4	-6.7	1.3
Residential mobility[a]	0.0	1.8	-0.1	1.3	0.1	4.5	4.6	3.7
ED visits prior to diagnosis	2.4	22.2	4.9	25.2	2.9	30.8	1.3	27.1
Primary care clinician visits	0.0	0.3	0.2	4.4	0.2	10.1	-1.1	17.3
Continuity of care[b]	0.6	3.5	1.9	-0.1	1.5	1.1	-0.7	-0.5
Comorbidity	1.9	1.5	0.7	5.3	0.1	2.0	-0.1	3.3

Abbreviations: UCC, urgent cancer care; ED, emergency department.

[a] Residential mobility includes postal code changes in the five years prior to diagnosis.

[b] Continuity of care included primary care clinician visits in the 6 to 30 months prior to diagnosis.

Notes: Excludes UCC and ED visits that were not during the eligibility period.

Table S4. Percent change in scaled Brier score from full model for UCC and ED visits by variable and follow-up period excluding sex from the model

	Follow-up period, % change							
	1 to 6 months		7 to 12 months		13 to 18 months		19 to 24 months	
Variable	UCC	ED	UCC	ED	UCC	ED	UCC	ED
Chemotherapy	76.2	1.8	62.8	5.9	55.2	3.0	92.7	6.4
Radiation therapy	323	2.1	0.4	1.0	4.7	1.0	13.6	2.4
Hormone therapy	3.5	2.1	4.4	3.3	2.4	-1.3	1.8	0.9
Immunotherapy	0.8	0.7	-0.1	0.7	-4.8	0.2	12.5	0.1
Cancer site	12.0	4.8	18.0	0.5	0.1	6.9	6.0	11.5
Stage	0.7	13.4	2.7	10.4	5.2	10.4	4.2	1.1
Age	4.4	0.1	2.3	-0.3	6.3	-4.2	7.3	-0.6
Income quintile	1.8	-0.1	0.4	5.3	-0.7	0.1	-6.5	1.3
Residential mobility[a]	0.0	1.8	-0.1	1.4	0.1	4.6	4.0	3.7
ED visits prior to diagnosis	2.4	22.3	5.5	25.1	2.9	30.5	1.4	27.2
Primary care clinician visits	0.0	0.4	0.2	5.0	0.2	10.0	-1.1	17.4
Continuity of Care[b]	0.6	3.5	2.1	-0.2	1.3	1.0	-0.8	-0.5
Comorbidity[b]	2.0	1.5	0.7	6.2	0.5	2.4	0.0	3.2

Abbreviations: UCC, urgent cancer care; ED, emergency department.

a Residential mobility includes postal code changes in the five years prior to diagnosis.

b Continuity of care included primary care clinician visits in the 6 to 30 months prior to diagnosis.

Table S5. Percent change in scaled Brier score from full model for ED visits by variable and follow-up period only during UCC hours of operation and including all reasons for an ED visit

Variable	During UCC hours	All reasons	During UCC hours	All reasons	During UCC hours	All reasons	During UCC hours	All reasons
	1 to 6 months		7 to 12 months		13 to 18 months		19 to 24 months	
Chemotherapy	1.8	1.7	6.9	4.9	4.2	3.1	2.7	5.0
Radiation therapy	2.8	2.6	3.0	0.9	0.8	0.9	8.1	2.3
Hormone therapy	2.2	2.0	2.4	3.3	-2.5	-1.1	3.5	0.7
Immunotherapy	0.3	0.7	4.4	0.9	0.3	0.3	-0.5	0.1
Cancer site	9.4	5.3	-1.3	1.7	4.2	5.5	7.3	9.9
Stage	15.1	15.1	3.6	7.7	7.0	8.4	3.3	1.1
Age	0.5	0.2	1.1	0.2	1.1	-4.1	3.0	1.0
Sex	0.0	-0.1	1.1	2.1	0.5	1.1	0.2	0.1
Income quintile	0.6	-0.1	3.9	5.5	0.7	0.6	2.9	1.7
Residential mobility[a]	0.9	1.7	2.5	1.4	5.2	4.2	4.2	4.4
ED visits prior to diagnosis	10.9	19.6	22.4	26.5	36.6	30.0	31.8	22.7
Primary care clinician visits	0.0	0.4	8.9	4.9	13.0	10.0	13.7	17.7
Continuity of care[b]	1.8	3.4	-0.1	0.0	-0.5	1.5	-0.7	-0.3
Comorbidity	2.8	1.2	1.6	4.6	2.2	2.2	-0.2	3.5

Abbreviations: ED, emergency department.

a Residential mobility includes postal code changes in the five years prior to diagnosis.

b Continuity of care included primary care clinician visits in the 6 to 30 months prior to diagnosis.

Figure S1. Description of when individuals diagnosed with cancer were included in the study cohort

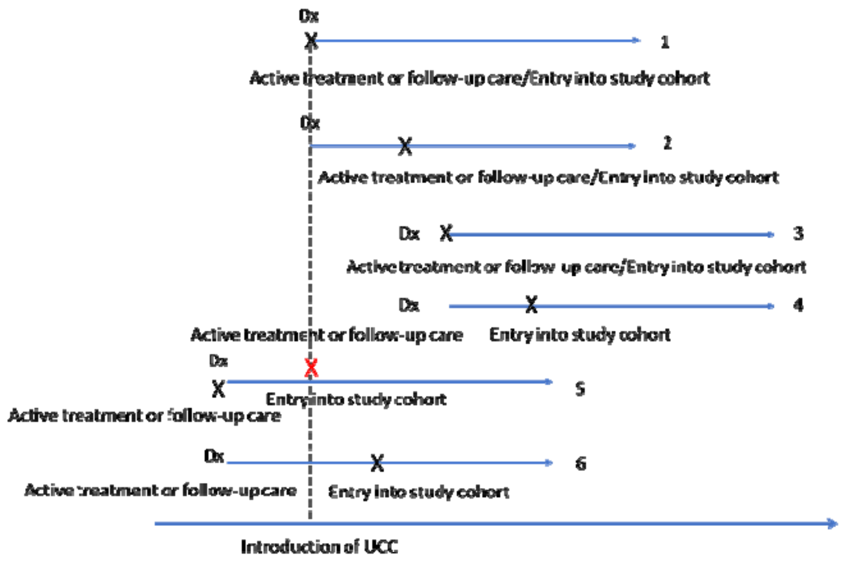

Dx; diagnosis date. X; date of entry into study cohort

1. Individuals who were diagnosed on November 4, 2013 and were eligible to attend the UCC on that day entered the study cohort on November 4, 2013. They contributed data to the study beginning at diagnosis.
2. Individuals who were diagnosed on November 4, 2013 but were eligible to attend the UCC at a later point in time entered the cohort on the date of UCC eligibility and only contributed data from that point forward.
3. Individuals diagnosed after the introduction of the UCC who were eligible to attend the UCC from diagnosis entered the study cohort at diagnosis.
4. Or when they became eligible to attend the UCC.
5. Individuals diagnosed prior to the introduction of the UCC entered the study cohort either on November 4, 2013.
6. Or when they became eligible to attend the UCC (example 6).

Figure S2. Predicted probabilities from a multivariable logistic mixed model describing the association of primary care clinician visits with ED visits stratified by follow-up time period (covariates held at their mean)

A.

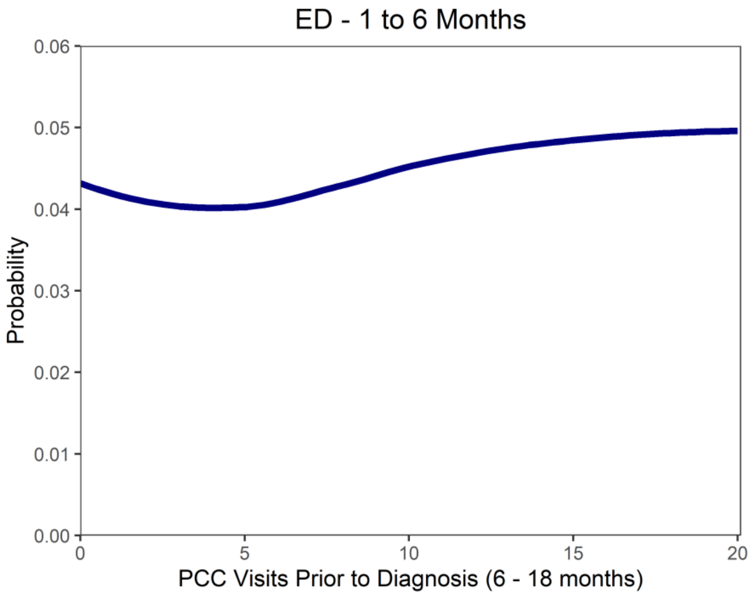

B.

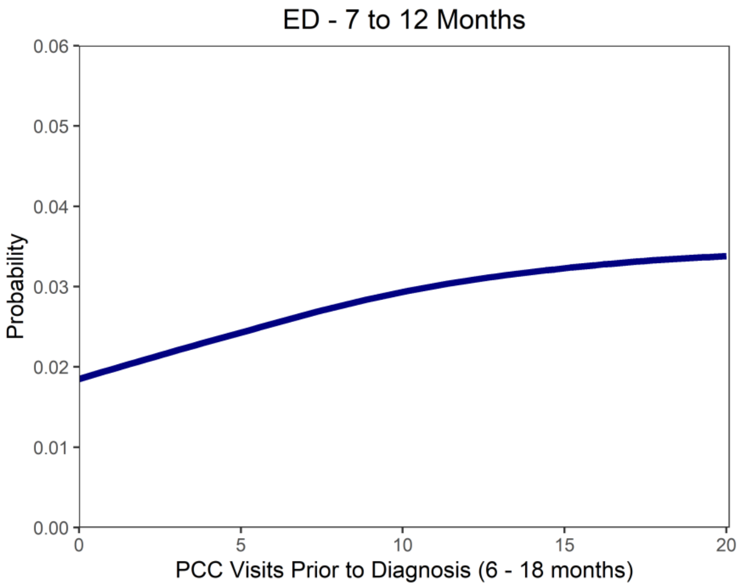

C.

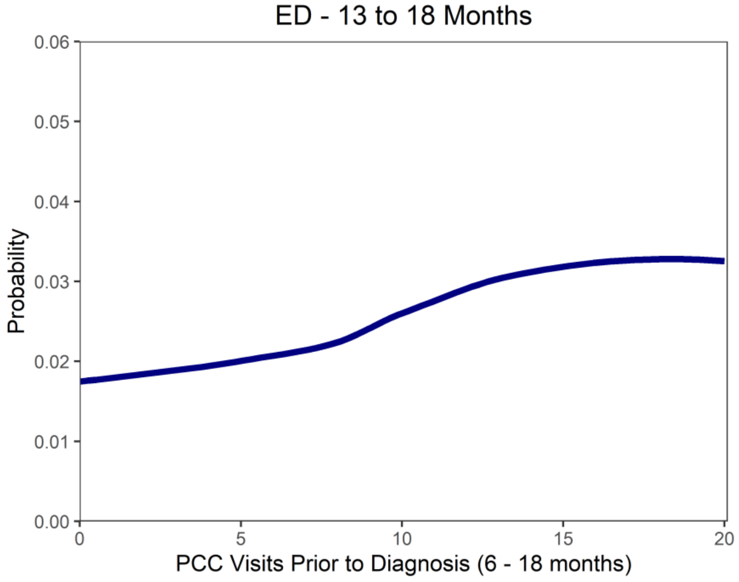
ED - 13 to 18 Months
Probability
0.06
0.05
0.04
0.03
0.02
0.01
0.00
0
5
10
15
20
PCC Visits Prior to Diagnosis (6 - 18 months)

D.

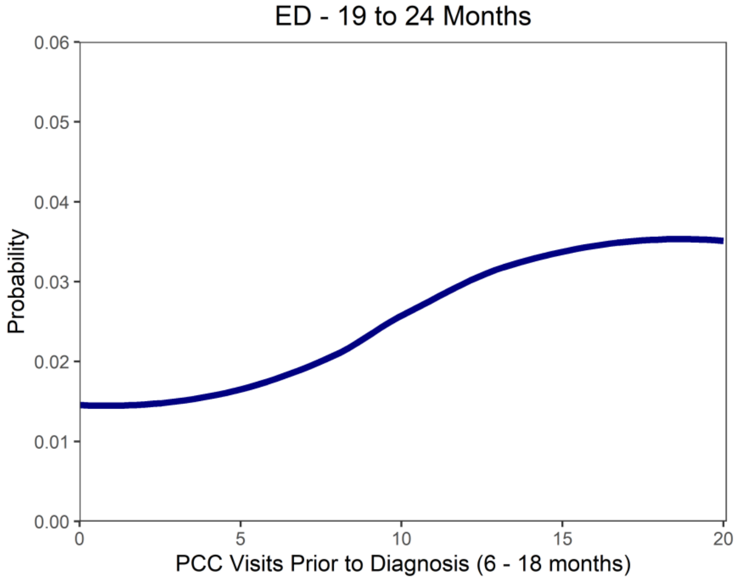
ED - 19 to 24 Months
Probability
0.06
0.05
0.04
0.03
0.02
0.01
0.00
0
5
10
15
20
PCC Visits Prior to Diagnosis (6 - 18 months)
